# Grand challenges for systems neuroscience: perspectives and opportunities

**DOI:** 10.3389/fnsys.2026.1818506

**Published:** 2026-04-07

**Authors:** Wen-Jun Gao

**Affiliations:** Department of Neurobiology and Anatomy, Drexel University College of Medicine, Philadelphia, PA, United States

**Keywords:** big data, grand challenge, new era, perspective, systems neuroscience

## Abstract

Systems neuroscience seeks to uncover how diverse neuronal populations and distributed neural types, circuits, and regions in the brain give rise to perception, learning, emotion, cognition, and behavior, and how dysfunction at any of these levels contributes to neurological and psychiatric disorders. The field has rapidly expanded from single-cell studies to large-scale, multimodal interrogation and manipulation of brain-wide activity, creating unprecedented opportunities alongside major conceptual and technical challenges. Systems neuroscience is at a pivotal moment, with advances in basic, translational, and clinical research converging to deepen understanding of how neural activity generates mental life.

## Introduction

Systems neuroscience is a subdiscipline of neuroscience that studies how neural networks at different levels (e.g., microcircuits, meso-networks, and macro-systems) integrate to produce complex functions such as sensation, emotion, cognition, and behavior. The field aims to elucidate the pathways of information flow within the central nervous system, establish causal relationships between coordinated neural activity and overall function, and investigate its association with neurological and mental disorders. The Mission and Scope of the Frontiers in Systems Neuroscience covers a wide range of systems neuroscience research, employing state-of-the-art methods and addressing diverse topics.^1^

The overarching “grand challenge” in systems neuroscience is to ultimately understand how the human brain functions and generates mental activity, thereby linking neural activity to perception, thought, emotion, and behavior, as supported by massive data from various studies. Systems neuroscience is now at a pivotal moment in integrating huge advances from basic, translational, and clinical neuroscience research. Over the past two decades, the field has moved from studying single neurons or small circuits toward observing and manipulating thousands to millions of cells across brain regions, integrating synaptic and cellular detail, and using computational frameworks that scale to behavior.

While this progress opens extraordinary opportunities, it also brings pressing conceptual and technical challenges. Despite the seemingly clear mission and scope, here I highlight the major challenges in this big data and artificial intelligence (AI) era. Below, I select some of the significant grand challenges facing systems neuroscience today, highlight recent advances that change the landscape, and sketch practical and scientific opportunities for the coming decade. Where appropriate, I cite representative recent work and reviews that exemplify these trends (Vogelstein et al., 2016; Altimus et al., 2020; Bader et al., 2025), especially those from publications of the Brain Initiative (Hawrylycz et al., 2023)^2^ and EBRAINS (Amunts et al., 2024),^3^ and I apologize for being unable to cite many critical discoveries or reviews.

## Great progress from single cells to whole circuits in behaving animals

There has been tremendous growth in new technologies that allow large-scale investigation of different features of the nervous system at unprecedented levels. A defining trend is the rapid increase in the scale, density, and chronic stability of recordings. Silicon electrode arrays (Neuropixels and successors) (Jun et al., 2017; Steinmetz et al., 2021; Ye et al., 2025), optical calcium imaging (de Vries et al., 2020; Zong et al., 2022) and high-frequency voltage “TEMPO” (transmembrane electrical measurements performed optically) dynamics (Haziza et al., 2025), and large-scale electrophysiology (Urai et al., 2022) now permit simultaneous single-unit resolution sampling of tens of thousands of neurons in rodents and have begun to be applied in non-human primates and human subjects. An emerging phenomenon in recent years is the use of combinations of these new techniques to determine direct links between different modalities (Arkhipov et al., 2025). These approaches dramatically improve the power to detect population-level motifs, dynamics, and even cell subtypes that underlie distinct neural function and behavior. For example, the major advance of the TEMPO technique is a critical development of an optical method that captures electrical brain waves up to roughly 100 Hz, enabling the observation of neural oscillations in freely moving mice in a cell-type- and pathway-specific manner, overcoming the shortcomings of *in vivo* recording (Haziza et al., 2025). Landmark demonstrations include large-scale single-neuron recordings in the human cortex using high-density probes and ongoing advances tailored to large-animal brains (Urai et al., 2022; Leonard et al., 2024). These techniques will provide the foundation for monumental breakthroughs in our understanding of how neural ensembles guide behavior (Jennings et al., 2019).

However, different technologies introduce distinct biases (e.g., optical sampling favors superficial layers; extracellular arrays undersample small neurons), complicating cross-study comparisons, especially across and within different species (Barron et al., 2021). There are still huge gaps between rodent, primate, and human studies. Rigorous benchmarks and widely adopted processing pipelines are needed, along with standards for metadata, so that large datasets become cumulative community resources. Importantly, interpreting and using the big data collected effectively poses a significant challenge. Investments in shared hardware standards, probe designs for chronic multi-area implants, and interoperable analysis toolkits will realize the promise of large-scale, reproducible experiments. Combining complementary modalities (e.g., Neuropixels + widefield imaging + behavior video) in standardized experimental batteries can create datasets that constrain theories in ways single-modality studies cannot (Urai et al., 2022; Hawrylycz et al., 2023). These and other technological hurdles are prioritized areas of the NIH Brain Initiative (US) website^4^ and https://www.braininitiative.org/.

## Linking structure to function and behavior with multiscale and multimodal connectomics

Neuroscience seeks to explain how the brain’s structural and functional organization gives rise to behavior. The development of neuroscience from Santiago Ramón y Cajal’s foundational “neuron theory” to modern systems behavior involves a shift from identifying individual, discrete nerve cells to understanding complex, dynamic, and interconnected networks. Cajal established that neurons are independent, polarized units that communicate via contacts (synapses), coined by physiologist Sir Charles Scott Sherrington (Molnár and Brown, 2010), setting the stage for neuroanatomy, physiology, and behavioral neuroscience (García-Lorenzo et al., 2025). After more than a century, mapping anatomy at the resolution required to explain function — synaptic connectivity across defined neuron types within behaving circuits — remains a challenge. Recent advances include functional connectomics efforts that pair large-scale functional recordings (Urai et al., 2022; Ramos-Llordén et al., 2026) with dense electron microscopy reconstructions (Velicky et al., 2023), producing datasets in which activity, morphology, and synaptic wiring are co-registered across hundreds of thousands of cells. For example, in the Fly brain, connections between neurons were mapped by analyzing electron-microscopic images, and amazingly, 93.3% of neurons are found to be in strongly connected networks (Dorkenwald et al., 2024), indicating massive input/output connections among all neurons. Incorporating this level of interconnectivity into computational models that link cell structure to function presents an additional challenge. While cellular-level anatomy provides the foundation for understanding function, molecular and functional data are also essential. At the mesoscopic scale, functional regions emerge from the collective activity of neuronal ensembles. Structure strongly constrains function at the microscopic level, but at meso- and macroscopic scales, understanding behavior requires accounting for complex, dynamic interactions across neural populations. Recognizing how structure–function relationships differ across scales is thus critical for developing accurate representational models (Constable, 2025).

Furthermore, recent advances in multiscale imaging have enabled detailed structural brain atlases, but these atlases alone cannot capture how function emerges from chemical modulation and flexible neural tuning within densely connected circuits. Functional organization arises from dynamic interactions layered on top of a fixed anatomical infrastructure, making cross-scale definitions of function difficult to reconcile. This highlights the need for careful integration of structural and functional perspectives—and clearer functional definitions across scales—to advance understanding of brain organization (Constable et al., 2026). Fortunately, such datasets are beginning to enable testing of how connectivity motifs give rise to population dynamics and computation (Hawrylycz et al., 2023; Ding et al., 2025).

Despite the grand progress, challenges remain. Perhaps the most critical challenge is to interpret the massive volume of high-dimensional descriptive map data using mechanistic models and interpretable theories. We must move beyond descriptive summaries toward mechanistic, interpretable computational models that predict neural dynamics and behavior. Powerful theoretical frameworks—dynamical systems, probabilistic population codes, and network-level modeling—offer candidate explanations for observed population motifs (Urai et al., 2022). Recent work shows that connectivity-informed network models can predict activity patterns, illustrating one path to mechanistic explanation (Arkhipov et al., 2025; Beiran and Litwin-Kumar, 2025).

Still, these challenges offer massive opportunities. For example, hybrid strategies—e.g., dense functional recordings in many animals and deep anatomical reconstruction in a smaller subset—can leverage statistical power while preserving mechanistic depth (Peng et al., 2021). Computational models that explicitly incorporate uncertainty from undersampling and inter-subject variability will be crucial (Jassim et al., 2025). Importantly, open, well-annotated connectomic resources will enable community-wide model testing and accelerate discovery. In addition, the development of standardized benchmarks (behavioral tasks, model evaluation metrics), causal perturbation experiments that test model predictions (chemogenetics, optogenetics, closed-loop stimulation), and tighter integration between experiment and theory can drive convergence (Wei et al., 2026). Encouragingly, community data challenges and shared datasets are starting to provide arenas for rigorous model comparison (Amunts et al., 2024). The brain research community worldwide has begun sharing databases to deepen understanding of the inner workings of the human mind and to improve how we treat, prevent, and cure brain disorders for the foreseeable future.

## Integrating molecular identity, cell types, and circuit dynamics

Systems neuroscience must reconcile the molecular diversity revealed by single-cell transcriptomics with circuit-level dynamics observed *in vivo*, although molecular neuroscience appears to be beyond the scope (Bonev et al., 2024). Single-cell and spatially resolved omics techniques now map transcriptional and epigenetic states with cellular resolution in intact tissue; when combined with physiology, these approaches offer exceptional opportunities to link gene expression to circuit roles, plasticity, and behaviors. Recent reviews outline opportunities and pitfalls of applying single-cell and spatial methods to neural systems (Hawrylycz et al., 2023; Bonev et al., 2024).

An obvious challenge in linking molecular signatures to electrophysiology and behavior is the need for reliable cell-type markers that generalize across animals and conditions. Recent single-cell projectome has presented a robust and efficient platform for imaging and reconstructing complete neuronal morphologies of thousands of neurons, including axonal arbors that span substantial portions of the brain from the mouse (Winnubst et al., 2019; Gao et al., 2022) and the primate (Xu et al., 2021; Gou et al., 2025). Amazingly, the reconstructed neurons could span more than 85 m of axonal length (Winnubst et al., 2019), and these detailed reconstructions have revealed previously unknown subtypes of projection neurons, suggesting organizational principles of long-range connectivity. Moreover, transcriptomic state can be transient and context-dependent. Experimental designs that dissociate stable cell identity from state-dependent changes (e.g., activity, neuromodulation, disease) are required. However, molecular readouts can guide genetically targeted perturbations, help explain interindividual variability, and illuminate mechanisms of plasticity and disease (Ng et al., 2024; Wei et al., 2026). Integrated multimodal pipelines (e.g., activity tagging followed by spatial transcriptomics) will be robust for causal inference and for building richer models of circuit function (Arkhipov et al., 2025).

## From neuronal function to richer, naturalistic, and ethological behavioral assays

Understanding brain function requires behavioral paradigms. Historically, constrained tasks offered experimental control but risked missing the complexity of natural behavior. Advances in video analysis and pose estimation, such as DeepLabCut (Mathis et al., 2018; Lauer et al., 2022),^5^ and in unsupervised behavior segmentation, enable detailed, quantifiable descriptions of naturalistic behavior at scale (Kennedy, 2022); integrating these behavioral descriptions with neural recordings is gradually becoming a routine.

However, richer behavioral data increase analytic complexity and raise the bar for causal inference. The multiplicity of behavioral measures leads to overfitting and spurious correlations unless theory-driven hypotheses or rigorous cross-validation are applied. Additionally, translating findings across species and task contexts require careful attention to ethological validity. Therefore, creating standardized, ethologically relevant behavioral batteries and developing interpretable behavioral embeddings will let systems neuroscience link neural computations to evolutionarily conserved functions. Fortunately, the Research Domain Criteria (RDoC) initiative at the NIH provides a set of research principles for investigating neuronal function and mental disorders through a biologically based approach rather than traditional symptom- or animal-model-based classifications. This initiative aims to foster new research approaches that will improve diagnosis, prevention, intervention, and treatment by integrating information from genetics, neuroscience, and behavioral science to explore fundamental dimensions of functioning that span the full range of human behavior, from normal to abnormal (Insel et al., 2010; Banaraki et al., 2024). As noted from the beginning, the RDoC is not intended to serve as a diagnostic guide or to replace current diagnostic systems. Instead, it aims to understand the nature of mental health and illness in terms of varying degrees of dysfunction in fundamental psychological/biological systems [Research Domain Criteria (RDoC) - National Institute of Mental Health (NIMH)].

## Data sharing, AI, translation, and ethics

Big science in systems neuroscience depends on shared data, standards, and reproducible pipelines. Historically, data sharing has been insufficiently pursued because the systemic incentives, technical infrastructures, and legal frameworks were originally constructed to support individual discovery and institutional property rather than communal access. The global research community is gradually navigating a pivotal transition toward an open science (National Academies of Sciences Engineering, and Medicine, Policy and Global Affairs, Board on Research Data and Information, and Committee on Toward an Open Science Enterprise, 2018) and a collaborative research ecosystem (Thibault et al., 2023). This shift is predicated on the foundational belief that scientific data, particularly when generated through public funding, represents a collective good with the potential to accelerate discovery, enhance reproducibility, and foster cross-disciplinary innovation. Community-led initiatives that define experimental metadata standards, common task descriptions, and open repositories have catalyzed progress in fields like genomics; neuroscience must accelerate analogous efforts. A great example, of course, and not limited to, is the huge dataset in the Allen Brain Atlas and EBRAINS (Amunts et al., 2024). A grand challenge now is how to use the data efficiently to guide future studies.

The current landscape suggests that while the benefits of sharing are widely acknowledged, the individual researcher often faces a net loss in terms of time, career security, and resources when attempting to make data truly Findable, Accessible, Interoperable, and Reusable (FAIR).^6^ Addressing these challenges requires a comprehensive overhaul of regulatory mandates, the deployment of privacy-enhancing technologies, and a fundamental realignment of institutional values, as exemplified by the Data Management and Sharing policy of the National Institutes of Health in the USA.^7^ Recently, the FAIR^2^ Data Management service, promoted by Frontiers, also provided researchers with tools to curate datasets, create an interactive FAIR^2^ Data Portal, and publish a peer-reviewed FAIR^2^ Data article, ensuring that research is reusable, citable, and ready for global impact.^8^

Recent advances in AI have drastically intensified the intersection between neuroscience and machine learning. AI is suddenly everywhere, offering tremendous opportunities and, at the same time, posing a huge challenge for overseeing AI in research and publications.

Systems neuroscience discoveries increasingly have translational reach: novel stimulation paradigms, brain–computer interfaces (BMIs), and biomarkers for brain disorders are moving toward clinical reality. The necessity of robust, interoperable data ecosystems is most evident in the rapidly advancing field of BMIs, where recent clinical applications have demonstrated the transformative potential of translating high-bandwidth neural data into functional outcomes. For example, Neuralink’s first human participant, Noland Arbaugh, has used the N1 implant to control a computer cursor with his mind, enabling him to browse the internet, play online chess, and play video games. Some recent successful clinical applications involve both sensory and motor systems. For example, natural thermal sensation has been restored in upper-limb amputees (Iberite et al., 2023), and precise tactile percepts have been evoked through multielectrode intracortical microstimulation of the somatosensory cortex (Greenspon et al., 2025) for sensory restoration. On the motor side, recent advances include Neuralink’s Noland case, Synchron’s COMMAND endovascular BCI trial for severe paralysis (Mitchell et al., 2023), and a high-performance intracortical BMI enabling finger-level decoding and quadcopter game control in an individual with paralysis (Willsey et al., 2025). These advances in BMIs provide promising clinical frontiers. The success of these systems relies on sophisticated AI decoders that require vast amounts of high-fidelity data to map neural intentions to sensory and motor actions (Maiseli et al., 2023; Zhang et al., 2024; Lavazza et al., 2025).

However, translating mechanistic insight into safe, equitable therapies requires careful ethical oversight, robust clinical trials, and attention to long-term effects. This has been, as always, a considerable challenge for systems neuroscience and, more generally, brain science. The interventions that alter neural dynamics can have complex, system-level consequences. The field must also address ethical questions around neuroprivacy, consent (particularly for vulnerable populations), equitable access to neurotechnology, and, importantly, AI use in scientific research and publications.

Integrating data sharing, AI, ethicists, clinicians, patients, and the public into research planning can both reduce risk and accelerate the societally beneficial translation of research. Systems neuroscience can responsibly shape neurotechnology policy by generating transparent evidence on benefits, risks, and limitations; thus, we must set the highest standards for systems neuroscience research and publications.

## Concluding perspective: coordinated, theory-driven acceleration

Systems Neuroscience is undergoing a rapid transformation, moving from fundamental, exploratory research to high-impact clinical, translational applications. This shift is driven by a convergence of advanced neurotechnologies, AI, and new, collaborative research models. The grand challenges for systems neuroscience are not purely technical but are universal both organizationally and conceptually. The field needs coordinated infrastructures (shared datasets and benchmarks), methodological rigor (standards for preprocessing, validation, and model comparison), and a commitment to mechanistic explanation grounded in testable predictions. Recent advances — from high-density physiological probes and functional connectomics to spatial omics and advanced computational and imaging methods, as well as the wise use of AI analyses — indicate the field is now capable of addressing questions that were previously out of reach. Realizing that promise requires interdisciplinary teams, open science, and a balance between ambitious large-scale projects and carefully controlled mechanistic experiments, as exemplified by the NIH Brain Initiatives (US) and many other large-scale brain projects worldwide.

If the community can align incentives, build shared infrastructure, and insist on rigorous hypothesis testing, the coming decade could yield a qualitatively deeper understanding of how neural circuits implement sensation, cognition, emotion, thinking, decision-making, and behavior, with clear implications for brain health and treatment of neurological and mental disorders. These are grand challenges, but they are tractable: tools are emerging, ideas are maturing, databases are increasing, and the opportunity to convert descriptive maps and datasets into mechanistic theories has never been greater.

## Data Availability

The original contributions presented in this study are included in this article/supplementary material, further inquiries can be directed to the corresponding author.

## Footnotes

1
https://www.frontiersin.org/journals/systems-neuroscience/about

2
https://www.braininitiative.org/alliance-resources/

3
https://ebrains.eu/

4
https://braininitiative.nih.gov/

5
https://github.com/DeepLabCut/DeepLabCut

6
https://www.go-fair.org/go-fair-initiative/

7
https://grants.nih.gov/grants/guide/notice-files/NOT-OD-21-013.html

8
https://www.frontiersin.org/about/fair-data-management
